# Supplementary material for: Transdermal Delivery of Metformin Using Dissolving Microneedles and Iontophoresis Patches for Browning Subcutaneous Adipose Tissue
Source: Pharmaceutics. 2022 Apr 17;14(4):879. doi: 10.3390/pharmaceutics14040879 (PMC9029293; doi:10.3390/pharmaceutics14040879)
Supplement: Supplementary file 1 [file pharmaceutics-14-00879-s001.zip › pharmaceutics-1644551-supplementary.pdf]

Supplementary Information

Supplementary Methods

Delivery effectiveness using the MN derma roller and iontophoresis (INT) ***in vitro***

For concept demonstration, *in vitro* permeation study using the MN derma roller and INT was first performed on the abdominal skin of pigs. Skin samples were cut with a scalpel into 5×5 cm^2 pieces, wrapped in foils and kept at -20°C until use.

The skin samples were taken from the freezer prior to the testsand thawed at room temperature for aroun d 20 minutes. After thawing, the skin surface was carefully wiped with cotton wool balls wetted with phosphate buffered saline 1X (1XPBS, pH 7.4) and the skin is then allowed to dry for around 5 minuets at room temperature. Titanium Micro Needle Facial Derma Roller Face Skin Massage Care Tool, with needle length of 0.5 and 1 mm was purchased from Amazon. Treatments were: 1. Control (no MN or INT), 2. MN derma roller (0.5 mm) alone, 3. MN derma roller (1 mm) alone, 4. INT alone (IOMED® electrodes with reservoir) (0.2 mA/cm^2, 30 minutes), and 5. MN derma roller (1 mm) + INT (IOMED® electrodes with reservoir) (0.2 mA/cm^2, 30 minutes). Using a gentile pressure, the MN derma roller was rolled each direction three times in four directions over the epidermal surface. In treatment 1, skin was topically treated with Rhod-PE dye solution (prepared by dissolving in deionized water at 0.005 M). In treatment 2 and 3, the skin was initially treated by the MN derma roller and then topically treated with the dye solution. In treatment 4, the solution was loaded into an iontophoretic patch and applied on the skin and in treatment 5, the solution was loaded into an iontophoretic patch and applied on the MN treated skin. Different time points; 2, 4, 6, 12 and 24 h were considered. Same amount of dye was used in all treatments. Prepared dye solution pipetted out and applied gently with a small stainless-steel spatula to the entire epidermal surface of each skin sample with extra care to ensure that no solution touched the sides of the skin samples. Each sample then placed separately with epidermal side facing up in petri dishes. Dishes covered with a lid, wrapped in foil, and kept for desired time intervals in an incubator maintained at a temperature of 37°C. After incubation, the skin samples were subsequently embedded into the optimal cutting temperature (O.C.T.) compound, then cut into 10-μm cross sections using a Leica CM1950 cryostat (Leica Biosystems, Buffalo Grove, IL, USA). Sectioned slides were imaged for fluorescent dye intensity in different skin layers and each time points using a fluorescence microscopy the EVOS® FL Auto Imaging System.

MN patch loading test

Loading capacity of the developed dye/drug-loaded MN patch was determined following 3 methods: 1. Recovery of loaded dye/drug released into the medium, 2. Recovery of loaded dye/drug delivered to the skin upon MN application, and 3. Mathematical calculation as detailed below:

1. Recovery of loaded DID/metformin released into the medium: *In vitro* release profiles in medium were tested using the DID/metformin-loaded MN patches, immersed in medium containing 1XPBS: ethanol (10:1, pH 7.4, 37°C). DID released from the MN patch was then analyzed for fluorescent intensity using a BioTek Synergy H1 plate reader. The amount of released dye recovered form medium was quantified based on the linear standard curve created with concentrations ranges 0.0039 – 1 mg/ml. The extracted metformin was measured by a reference high-performance liquid chromatography (HPLC) method with modification (Waters HPLC system equipped with 1525 binary pump, 2707 autosampler with refrigeration unit, 2489 dual wavelength UV-visible detector, 2475 multi λ fluorescence detector, 2465 electrochemical detector and in-line degasser, with ZORBAX SB-C18, 4.6 × 250 mm, 5-micron column). The mobile phase was composed of acetonitrile and 1XPBS (60:40, v/v), a flow rate was 1 ml/min, and UV detection wavelength was 236 nm and quantified based on the linear standard curve created with various known concentrations [1].

2. Recovery of loaded DID/metformin delivered to the skin upon PLGA MN application:

2.1. DID/metformin-loaded PLGA MN patch was inserted into pig skin using thumb pressing and left in situ for 24 h. After removal of the MN patch, the punctured area was tape-stripped (Science ware® Write-On™ Label Tape) twice to remove the residual dye/drug left on the skin surface. Both the inserted MN patch and stripped tapes were soaked in medium containing 1XPBS: ethanol (10:1, pH 7.4, 37°C) to recover the dye/ drug. The extracted solution was then analyzed using plate reader or HPLC methods as described above. The amount of DID/metformin delivered into the skin was calculated by subtracting the amount of drug remaining in the MN patch after insertion and on the skin surface (recovered from dissolution testing in medium as described in 1) from the amount initially loaded in the non-inserted MN patches (quantified as described in 1).

2.2. Metformin-loaded PLGA MN treated skin sample was homogenized in 1 ml of saline (0.9% sodium chloride). After vortexing for 5 minutes, 1 ml of acetonitrile was added into the mixture. After vortexing the above mixture followed by centrifugation at 3,000 xg and 4℃ for 10 minutes, the upper aqueous phase was transferred into a new tube. Another 1 ml of acetonitrile was added to the bottom phase to repeat extraction. The combined acetonitrile was dried under nitrogen gas. The dried drug was reconstituted by saline and subsequently transferred in a HPLC vial for determining metformin concentrations using the above mentioned HPLC method.

3. Mathematical calculation: The loading capacity of MN was estimated using mathematical calculation based on MN geometry and number of needles per patch as below:

Square Pyramid Shape

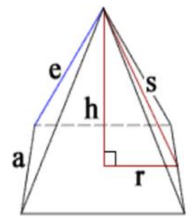

h = height
s = slant height
a = side length
e = lateral edge length
r = a/2
V = volume
L = lateral surface area
B = base surface area
A = total surface area

<u>Company provided mold size:</u>
a= 0.2 mm
h= 0.8 mm
B=a^2=0.04 mm^2
Volume=1/3*B*h= 0.0106 mm^3
Volume of MN patch with 100 needles= 0.0106 mm^3 * 100 = 1.06 mm^3
mg/ mm^3
mass≈106 μg

<u>SEM measured size of fabricated MN patch:</u>
a= 0.2 mm
h= 0.765 mm
Volume=1/3*B*h= 0.0102 mm^3
Volume of MN patch with 100 needles= 0.0102 mm^3 * 100 = 1.02mm^3
mg/ mm^3
mass≈102 μg

Release profile of the metformin-loaded MN patch *in vitro*

The metformin-loaded MN patch immersed in 1XPBS: ethanol (10:1, pH 7.4, 37°C). Released metformin into the medium was collected at different time intervals (replaced with fresh medium) and analyzed using a reference high-performance liquid chromatography (HPLC) method (Waters HPLC system, with ZORBAX SB-C18, 4.6 × 250 mm, 5-micron column). The mobile phase was composed of acetonitrile and 1XPBS (60:40, v/v), a flow rate was 1 ml/min, and UV detection wavelength was 236 nm and quantified based on the linear standard curve created with various known concentrations [1].

Metformin stability in the dissolvable MN patches

Four MN patches loaded with 92 μg metformin were stored at 4°C for 7 days. After 7 days of storage, the remaining amount of metformin was quantified by dissolving MN patch in dissolution medium and measured using the above mentioned HPLC method.

***In vitro* transdermal delivery of dye using a MN patch and INT**

The skin samples were prepared as in "Delivery effectiveness using the MN derma roller and INT *ex vivo*". DID-loaded MN patch was fabricated as described in "Fabrication of the MN patch". The abdominal pig skins were treated as follows: 1. Control (No MN or INT), 2. INT alone (IOMED® electrodes with reservoir) (at 0.2 mA/cm^2, 30 minutes), 3. MN alone 24 h, and 4. MN + INT24 h (TENS electrodes without reservoir) (at 0.2 mA/cm^2, 30 minutes). In treatment 1, skin was topically treated (as in MN derma roller) with the dye solution (the same concentration and amount that was loaded into the MN patch (30 μl of 0.005 M dye dissolving in deionized water). In treatment 2, PLGA MN patch loaded with the same amount dye was pushed into skin by thumb-pressing the backing layer of MN patch and holding it for ~30 seconds. The MN patch was left in situ at 37°C for 2 and 24 h. In treatment 3, after treating the pig skins for 24 h as described in the treatment 2, the MN patch was removed, and INT was immediately applied at 0.2 mA/cm^2, for 30 minutes.

Supplementary Results

In vitro transdermal delivery of dye using the MN derma roller and INT

Permeation of the applied Rhod-PE dye through the skin layers was seen from epidermis to the subcutaneous WAT. After 2 h of treatments (Figure. S1, A to E); control had the strongest intensity of the dye on the epidermis layer, while using the MN derma roller improved the permeation through the layers and the fluorescent intensity on the epidermis was lower than the control. Dye permeation was similar for INT alone or MN derma roller alone treatments. However, the combination of the MN derma roller and INT greatly enhanced the dye permeation to the subcutaneous WAT. Even after only 2 h, we saw strong dye signals in the dermis and subcutaneous WAT and the intensity of the dye on the epidermis is weak (Figure. S1 E). The same trend was seen for the rest of time points. Dye permeation from epidermis to dermis and subcutaneous WAT was time-dependent in all treatments. Considering the 24 h time point, the skin treatment with combined MN derma roller and INT had the strongest signals in the subcutaneous WAT among all treatments. Therefore, the dye permeation was positively correlated with increment of time in all treatments. Combined MN derma roller and INT treatments significantly enhanced the permeation rate of Rhod dye across skin layers to subcutaneous WAT. It is noted that these results are for pig skins which are much thicker than those of mice or human beings.

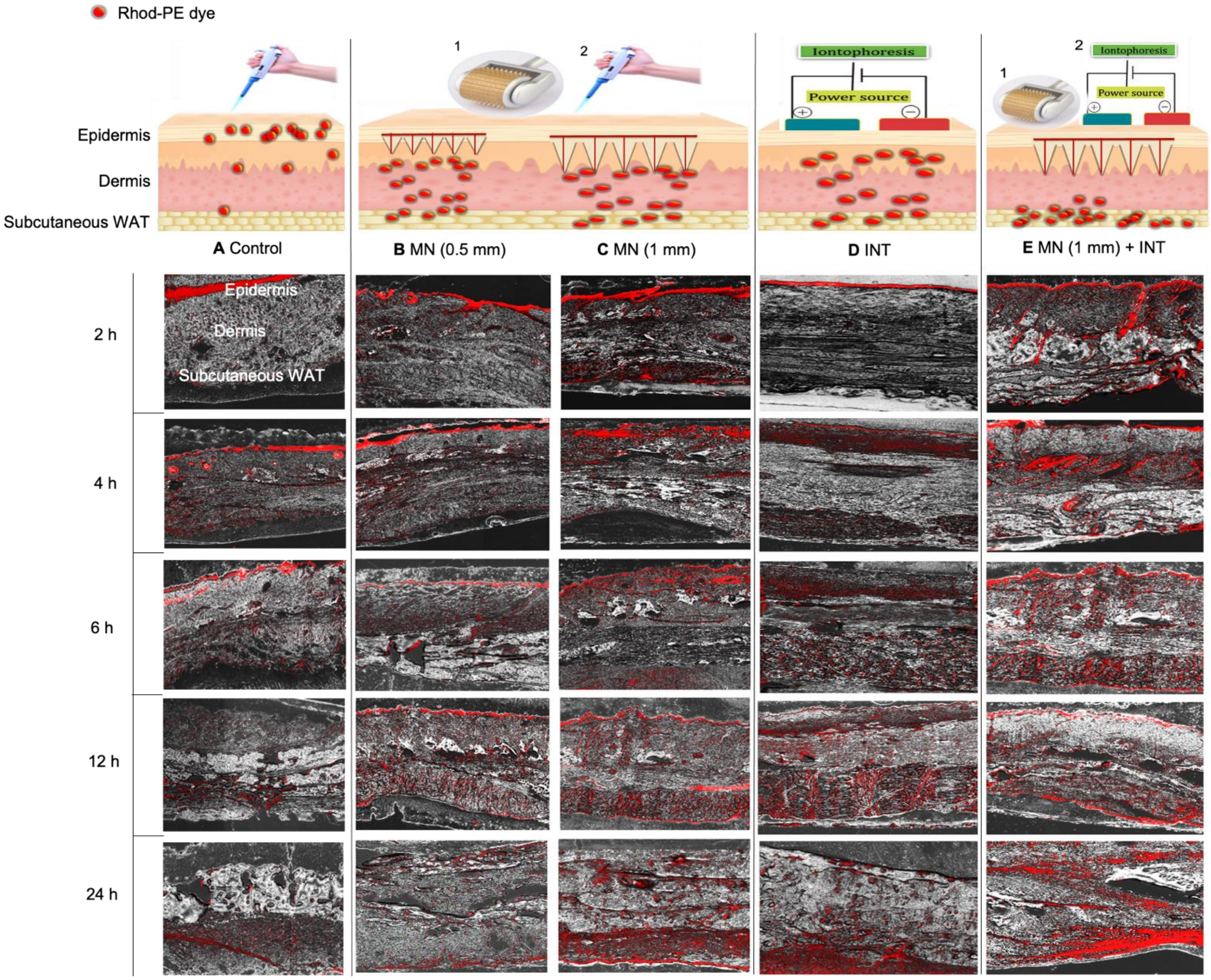

Figure S1. ***In vitro*** **transdermal delivery of Rhod-PE dye using MN derma roller and INT at different time points.** (**A**) Control (No MN or INT), (**B**) MN (0.5 mm), (**C**) MN (1 mm), (**D**) INT, (**E**) MN (1 mm) + INT.

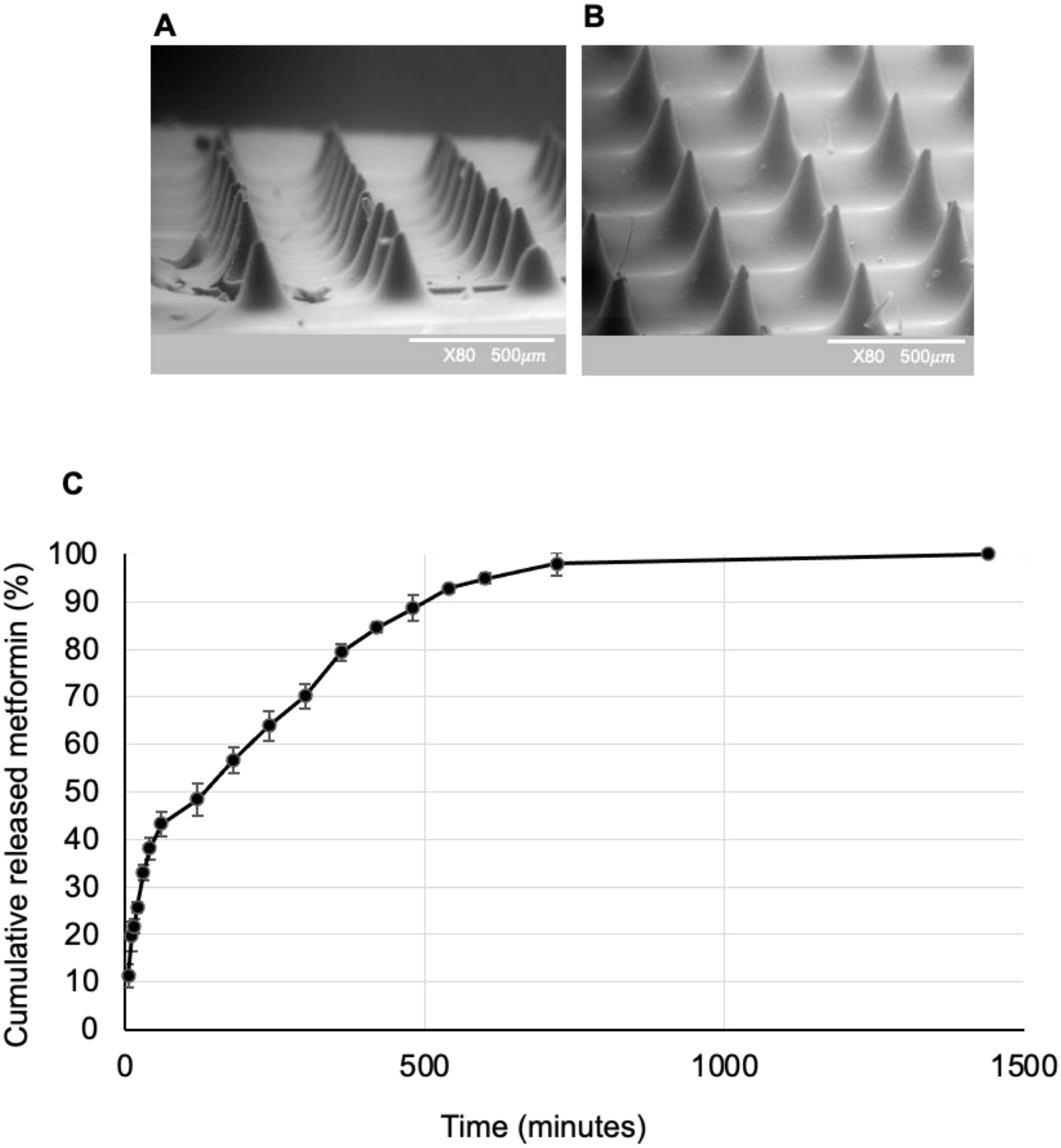

Figure S2. Microneedle charachteristics. (**A**) 800 μm PLGA MN after skin insertion 24 h (SEM view: 10° vertical), (**B**) 800 μm PLGA MN after skin insertion 24 h (SEM view: 60° vertical) (**C**) Metformin-loaded MN patch release profile.

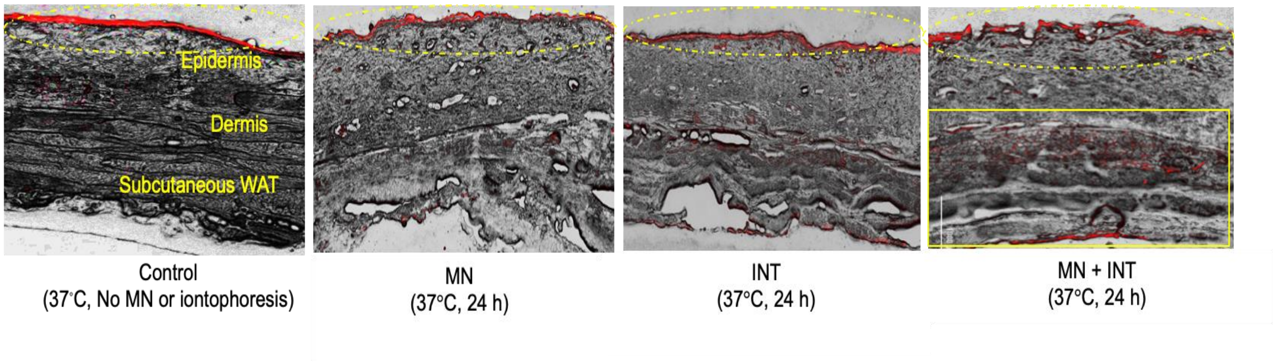

Figure S3. *In vitro* transdermal delivery of dye using PLGA MN and INT using pig skin.

Table S1. Metformin stability in MN patches

MN Patch	Expected initial loaded content (µg)	Recovered after 7 days (µg)	%
1	92	91.06	98.98
2	92	89.3	97.07
3	92	89.02	96.76
4	92	91.38	99.33
Average	-	90.19	98.033
SEM	-	1.2	1.3

Table S2. Primer sequences

36B4	Forward	GCTTCGTGTTCACCAAGGAGGA
	Reverse	GTCCTAGACCAGTGTTCTGAGC
β-actin	Forward	GTGACGTTGACATCCGTAAAGA
	Reverse	GCCGGACTCATCGTACTCC
UCP1	Forward	GCTTTGCCTCACTCAGGATTGG
	Reverse	CCAATGAACACTGCCACACCTC
PGC-1α	Forward	GAATCAAGCCACTACAGACACCG
	Reverse	CATCCCTCTTGAGCCTTTCGTG
PPARγ	Forward	GTACTGTCGGTTTCAGAAGTGCC
	Reverse	ATCTCCGCCAACAGCTTCTCCT
PRDM16	Forward	ATCCACAGCACGGTGAAGCCAT
	Reverse	ACATCTGCCCACAGTCCTTGCA
IL6	Forward	GTTCTCTGGAAATCGTGGA
	Reverse	GCCACTCCTTCTGTGACTCC
TMEM26	Forward	ACCCTGTCATCCCACAGAG
	Reverse	TGTTTGGTGGAGTCCTAAGGTC
ELOVL3	Forward	ACAGAGGCACACACAAACAC
	Reverse	GATAGGGAAGCAGGGTCTCC
Leptin	Forward	TGGGGTTTTGGAGCAGTTTG
	Reverse	CTGTCACTCTTTCCCGGTCT
CIDEA	Forward	GGTGGACACAGAGGAGTTCTTTC
	Reverse	CGAAGGTGACTCTGGCTATCC
MCP1	Forward	TCGCTCAGCCAGATGCAAT
	Reverse	ATCTCCTTGGCCACAATGGTC
GLUT2	Forward	TGAGTTCCTTCCAGTTCGGC
	Reverse	CTGAGGCCAGCAATTCTGACT
PI3K	Forward	ACACCAACGGTTTGGACTATGG
	Reverse	GGCTACAGTTAGTGGGCTTGG
NRF1	Forward	GCAGCACCTTTGGAGAATGTG
	Reverse	AATTAACCTCCTGTGGCGCAG
GLUT4	Forward	GCTCTGACGTAAGGATGGGG
	Reverse	TGGCCAGTTGGTTGAGTGTT
PEPCK	Forward	AATGCTTTCTCAAAGTCCTC
	Reverse	AATATGACCAACTGTTGGCTG
G6P	Forward	AGATAGCAAGAGTAGAAGTGAC
	Reverse	TTCCAAGTGGATTCTGTTTGG
AKT	Forward	ATGAACCGACGACGTAGCCATTGTG
	Reverse	TTGTAGCCAATAAAGGTGCCAT
TNF$\boldsymbol{\alpha}$	Forward	CCTGTAGCCCACGTCGTAG
	Reverse	GGGAGTAGACAAGGTACAACCC

Reference

1. Porta V, Schramm SG, Kano EK, Koono EE, Armando YP, Fukuda K, et al. HPLC-UV determination of metformin in human plasma for application in pharmacokinetics and bioequivalence studies. J Pharm Biomed Anal. 2008;46(1):143-7.
